# The Irony of Iron Pill Gastritis: A Case of Delayed Recognition and Persistent Injury in an Elderly Patient

**DOI:** 10.7759/cureus.71565

**Published:** 2024-10-15

**Authors:** Charles R Lichtenstern, Naeem Akhtar

**Affiliations:** 1 Department of Biomedical Education, California Health Sciences University College of Osteopathic Medicine, Clovis, USA; 2 Department of Gastroenterology, California Gastroenterology Associates, Fresno, USA

**Keywords:** case report, esophagogastroduodenoscopy (egd), ferrous sulfate, gastritis, gastroesophageal reflux disease (gerd), iron pill gastritis

## Abstract

Iron pill gastritis is an underrecognized complication of oral solid iron supplementation, particularly in elderly patients. We present the case of an 83-year-old male with a history of hypertension, hyperlipidemia, type II diabetes, and chronic gastroesophageal reflux disease who developed gastric ulcers and erosions after three months of ferrous sulfate therapy for iron deficiency anemia. Esophagogastroduodenoscopy revealed nonbleeding ulcers and erosions, and biopsies confirmed iron pill gastritis. Despite recommendations to switch to liquid iron, the patient continued using solid tablets, resulting in persistent mucosal irritation. This case highlights the importance of early recognition, patient education, and consideration of liquid iron formulations in at-risk populations.

## Introduction

Iron pill gastritis is an underrecognized disease but a relatively common complication of oral iron supplementation, typically observed during the treatment of iron deficiency anemia. It is well established that iron can lead to numerous gastrointestinal (GI) side effects, including nausea, constipation, diarrhea, blackened stools, and changes to the gut microbiome [1,2]. Daily use of ferrous sulfate was linked to a 2.6-fold increase in the likelihood of experiencing GI side effects compared with placebo or intravenous iron [1]. However, while these side effects are often manageable, gastric ulceration, on the other hand, is uncommon but can be a serious complication [3,4]. Notably, a 2006 study reported that the mean age of patients with iron-induced erosive injury was 76 years, underscoring the vulnerability of older adults [5]. Additionally, a 2008 study demonstrated that 98% of cases of iron deposition were linked to oral iron intake [6]. Given the high prevalence of iron deficiency anemia in the geriatric population, physicians should remain vigilant for the potential adverse effects of iron therapy on the upper GI tract.

We present the case of an 83-year-old male who presented to a rural outpatient gastroenterology clinic with nonspecific abdominal pain and loose stools. Esophagogastroduodenoscopy (EGD) revealed multiple nonbleeding ulcers in the gastric body and antrum and several small erosions with stigmata of recent bleeding. Biopsies confirmed iron deposition, consistent with a diagnosis of iron pill gastritis.

## Case presentation

**Figure 1:**
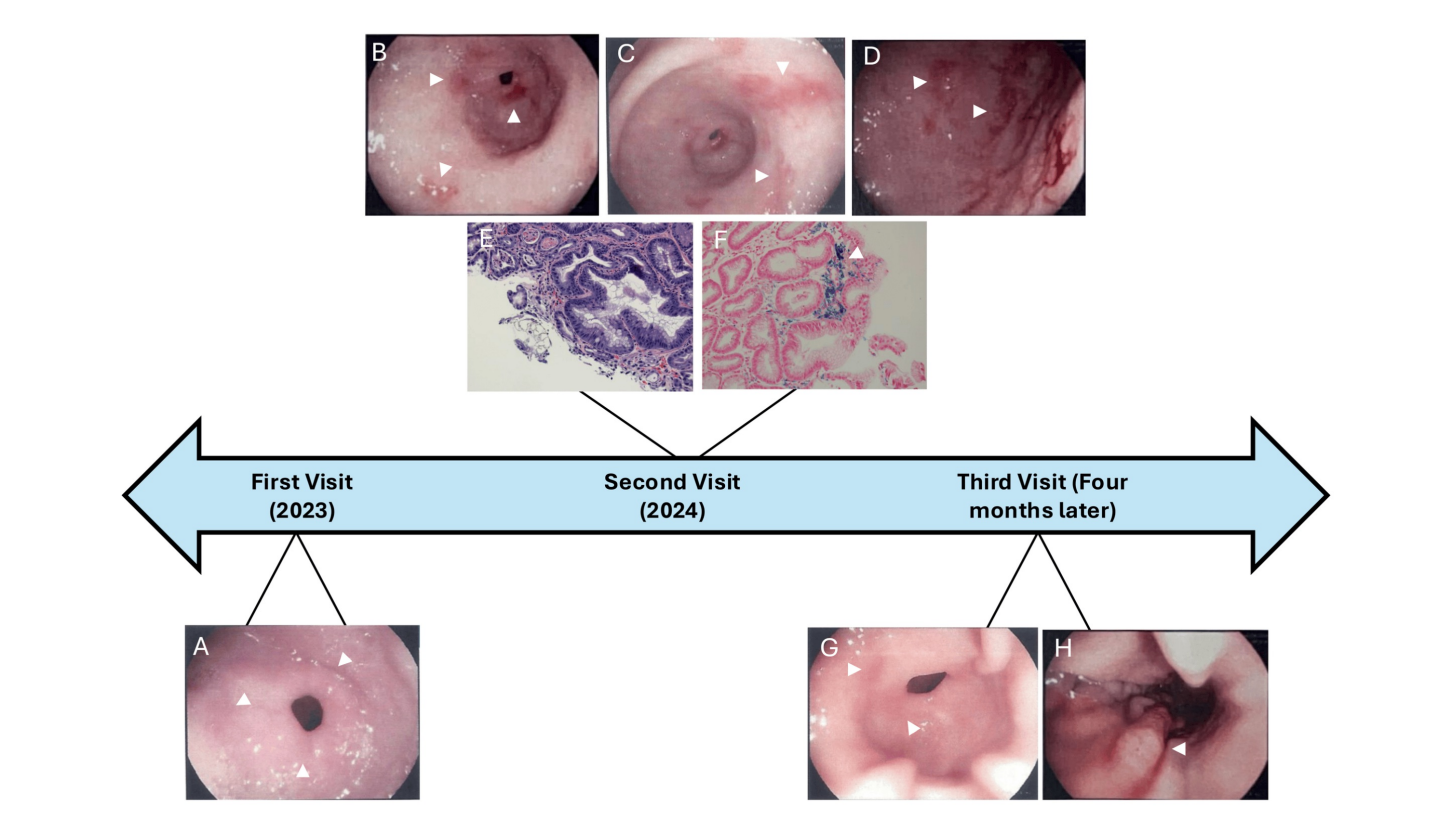
Esophagogastroduodenoscopy and biopsy images of the gastric mucosa. (A) Patchy, moderately erythematous mucosa without bleeding at the prepyloric region of the stomach. (B) Three nonbleeding ulcers at the prepyloric region of the stomach. (C) Two linear erosions at the prepyloric region of the stomach. (D) Erosion with stigmata of recent bleeding. (E) Hematoxylin and eosin stain of the gastric mucosa. (F) Giemsa iron stain of the gastric mucosa showing iron deposition. (G) Patchy, moderately erythematous mucosa without bleeding in the prepyloric region of the stomach. (H) Healing of previously noted gastritis

An 83 year-old-male patient presented by referral from his primary care physician (PCP) to a GI clinic for abdominal pain, diarrhea, and weight loss. The patient had a medical history of hypertension, hyperlipidemia, type II diabetes mellitus, and chronic gastroesophageal reflux disease (GERD). Medications included multiple courses of omeprazole, metformin, and a long history of nonsteroidal anti-inflammatory drug (NSAID) use for pain. Physical examination revealed epigastric abdominal tenderness. Given the concern for peptic ulcer disease (PUD) or celiac disease, an EGD was performed, which revealed patchy, moderately erythematous mucosa in the gastric antrum without bleeding (Figure 1A). The examined duodenum was normal. The mucosa was biopsied, showing mild reactive gastropathy of the antrum. The patient was treated conservatively with antireflux measures, including refraining from eating two to four hours before sleeping, elevating the head of the bed 6-10 inches, avoiding precipitating foods (e.g., greasy, spicy, and acidic foods, chocolate, and coffee), dividing meals into smaller quantities, and continuation of his omeprazole.

The patient presented one year later for abdominal pain and loose stools. Physical examination was unremarkable. A second EGD was performed, which revealed three nonbleeding superficial gastric ulcers in the gastric body and prepyloric region of the stomach and a few dispersed small erosions with stigmata of recent bleeding in the gastric body and antrum (Figures 1B-1D). The largest lesion was 10 mm in dimension. The ulcers and mucosa were biopsied, showing iron pill gastritis in the antrum and mild inactive chronic gastritis with reactive gastropathy in the prepyloric region (Figures 1E, 1F). The patient was advised to continue antireflux measures, omeprazole, and abstinence from NSAIDs. After-visit discussion with the patient revealed that his PCP had ordered routine lab work, which revealed iron deficiency anemia. The patient was being managed for the anemia with 325 mg of ferrous sulfate by mouth twice daily. At the time of the second EGD, the patient had been on ferrous sulfate for about three months. The patient was advised to speak with his PCP about switching from a solid tablet to a liquid preparation.

The patient presented four months later for a follow-up after being diagnosed with iron pill gastritis. The patient denied any complaints but still reported taking oral ferrous sulfate. Physical examination was unremarkable. A third EGD was performed to reevaluate the iron pill gastritis and ulcers and assess for healing, which revealed patchy moderately erythematous mucosa in the gastric body and antrum without bleeding, as well as healing of the previously noticed gastritis and ulcers (Figures 1G, 1H). A mucosal biopsy revealed reactive gastropathy of the gastric antrum with iron deposits, which is still consistent with iron pill gastritis. The patient was switched to famotidine and advised to continue antireflux measures. The patient was again advised to talk with his PCP to switch from solid iron to a liquid-based preparation.

## Discussion

This case highlights the underrecognized yet clinically significant condition of iron pill gastritis, a complication of oral iron supplementation that can lead to mucosal injury in the upper GI tract. Iron deposition in the gastric mucosa, as seen in this patient, results in both erosive and ulcerative lesions, which can complicate the management of iron deficiency anemia, particularly in older patients [5-7].

In this case, the patient had been taking ferrous sulfate for only three months before developing significant gastric erosions and ulcers, emphasizing how quickly iron-induced GI injury can occur. The short time frame in which the patient developed iron pill gastritis supports prior literature suggesting that iron-induced damage can manifest relatively quickly, especially in individuals with preexisting GI conditions, such as chronic GERD in this patient [8,9]. This reinforces the need for physicians to be vigilant when prescribing iron supplements to patients with underlying GI conditions, particularly those already on medications such as NSAIDs and proton pump inhibitors, which can exacerbate or mask mucosal injury.

The lack of communication between the patient's PCP and gastroenterologist could have delayed the identification of iron pill gastritis. The patient did not initially disclose his use of iron supplements until after the diagnosis was made, complicating the clinical picture. This case underscores the importance of clear and open communication between PCPs, specialists, and patients to ensure that all contributing factors, including medications, are considered during the diagnostic process. A simple discussion between physicians might have prompted earlier consideration of iron pill gastritis as a potential cause of the patient's symptoms, possibly mitigating further GI damage.

Given the patient's age and underlying medical conditions, a switch from oral iron tablets to a liquid preparation was recommended. Liquid iron formulations are often better tolerated and less likely to cause mucosal injury, particularly in elderly patients with prior GI disease, due to an inability to concentrate within the stomach to the extent of solid iron [2,7,10-12]. This recommendation aligns with general clinical guidelines, which suggest that liquid or intravenous iron should be considered in patients at risk for iron pill gastritis, especially in those with a history of PUD, GERD, or those taking concurrent NSAIDs [13]. Unfortunately, despite multiple advisements, the patient continued taking ferrous sulfate tablets, leading to the persistence of mucosal irritation on follow-up endoscopy. This further highlights the need for patient education and closer follow-up to ensure adherence to treatment recommendations and avoidance of preventable complications.

In discussing the potential advantages of liquid iron supplements, it is important to consider current recommendations and practices surrounding oral iron therapy. While liquid iron may pose a lower risk for certain GI side effects, such as gastritis, particularly in at-risk populations, the American Gastroenterological Association guidelines do not express a preference for liquid formulations over solid ones. All forms of iron supplementation carry a risk of GI side effects, though gastritis is more commonly associated with solid oral iron. Liquid iron is typically prescribed only when solid forms cause adverse effects. Furthermore, current evidence does not show a clear superiority of one formulation over the other in terms of efficacy or overall tolerability [13]. Additionally, solid iron preparations tend to be more cost-effective, likely contributing to their continued use as the standard of care. Given the absence of clear advantages and the economic considerations, solid oral iron remains the predominant choice in clinical practice. Future studies should explore whether liquid iron formulations offer a more favorable side effect profile or improved patient outcomes compared to solid preparations, particularly in the geriatric population.

## Conclusions

In conclusion, iron pill gastritis is a condition that requires early recognition and intervention. The use of liquid iron preparations should be strongly considered in elderly patients, especially those with a history of GI disorders. Improved communication between physicians and patients is essential in preventing and managing this condition effectively, ensuring that treatment decisions are made collaboratively and with all relevant information.
